# AHP-MOORA framework for longitudinal evaluation of Pharm.D program learning outcomes: a tool for Saudi pharmacy programs accreditation and curriculum enhancement

**DOI:** 10.3389/fmed.2025.1628510

**Published:** 2025-08-06

**Authors:** Sultan M. Alshahrani

**Affiliations:** Department of Clinical Pharmacy, College of Pharmacy, King Khalid University, Abha, Saudi Arabia

**Keywords:** pharmacy education, program learning outcomes, NCAAA, quality assurance, Saudi Arabia, Pharm.D

## Abstract

**Introduction:**

As pharmacy education in Saudi Arabia progresses in accordance with Saudi Vision 2030 and international competency-based standards, the necessity for systematic evaluation of Program Learning Outcomes (PLOs) has become essential. This study aimed to establish a scalable framework for evaluating PLOs’ achievement under the National Commission for Academic Accreditation and Assessment (NCAAA) criteria.

**Methods:**

A mixed-methods methodology was utilized in a public pharmacy institution in Saudi Arabia. Eight PLOs derived from the Pharm.D program framework were assessed utilizing stakeholder feedback and national quality standards. The analytical hierarchy process (AHP) was employed to prioritize outcomes, while the Multi-Objective Optimization by Ratio Analysis (MOORA) technique was utilized to evaluate performance over five graduating cohorts (2018–2022). Data sources comprised alumni employment data, postgraduate enrollment, and professional practice positions. A program based on Microsoft Access was created for data administration and visualization.

**Results:**

PLO2 (clinical application) and PLO5 (ethics and professionalism) earned the greatest AHP weights, measuring 0.28 and 0.19, respectively. Composite MOORA scores exhibited optimal performance in 2020 (0.883) and declined to 0.792 in 2022. The decline in 2022 may be attributed to reduced clinical training opportunities and delayed workforce absorption following COVID-19-related disruptions, as documented in comparable educational settings. The technology facilitated long-term trend analysis and cohort-specific reporting.

**Conclusion:**

The holistic monitoring tool established a durable framework for evaluating PLOs attainment by national accreditation standards. It facilitated evidence-based enhancements to the curriculum and enhanced institutional quality assurance. The widespread application of this strategy in pharmacy colleges, including digital integration and external benchmarking, can increase accreditation preparedness and facilitate ongoing educational enhancement.

## Introduction

Over the last twenty years, higher education in Saudi Arabia has experienced significant reforms to conform to international academic norms and national development objectives. The revisions were primarily motivated by Saudi Vision 2030, which prioritizes human capital development and a knowledge-based economy (1). The National Commission for Academic Accreditation and Assessment (NCAAA), presently operating under the Education and Training Evaluation Commission (ETEC), was assigned the responsibility of supervising quality assurance in higher education as part of this national transition (2).

Pharmacy education has experienced substantial transformation during this period of time. Originally concentrated on pharmaceutical sciences following the founding of the inaugural college in 1959, the paradigm began to evolve in the early 2000s, prioritizing clinical competencies, patient care, and experiential education. The initiation of Saudi Arabia’s inaugural Doctor of Pharmacy (Pharm.D) program at King Abdulaziz University in 2002 signified a crucial transformation in contemporary pharmacy education (3). This transition indicated a worldwide trend in pharmacy education towards clinical and community-focused jobs (4).

Saudi Vision 2030’s emphasis on transforming healthcare and education has directly impacted pharmacy education by promoting outcome-based learning, clinical role preparation, and integration of digital health competencies. These reforms have accelerated the development of Pharm.D programs that align with labor market needs, accreditation standards, and national workforce priorities. Further, the vision prioritizes the transformation of healthcare, the development of human capital, and education focused on outcomes. Pharmacy colleges must now conform to the Saudi Qualifications Framework (SAQF) and get national accreditation from the NCAAA and ETEC, indicating a systemic transition to competency-based standards (1, 5, 6).

Institutions are therefore under growing pressure to implement data-driven quality assurance systems and monitor graduate preparedness in a variety of professional practice domains.

With the increasing number of pharmacy programs throughout the Kingdom. The necessity for a uniform, outcomes-oriented quality framework became apparent. Inconsistencies in curriculum design, evaluation, and graduation preparedness underscored the lack of unified quality assurance systems (5). As a result, accreditation, which was previously voluntary, became mandatory for all academic programs under the auspices of the NCAAA. Accreditation emphasizes the alignment of program objectives with institutional missions, the relevance of the curriculum, and the assessment of program learning outcomes (PLOs) (6).

The accreditation process in pharmacy education evaluates essential areas like instructional design, faculty qualifications, infrastructure, clinical training, and graduation outcomes (7). The Saudi Arabian Qualifications Framework (SAQF) establishes a systematic basis for delineating and correlating learning outcomes in terms of knowledge, skills, and competencies. Programs must record and assess results such as employment rates, leadership positions, and postgraduate enrollment as indicators of their efficacy (8).

Institutions that achieved NCAAA accreditation indicated enhancements in educational planning, teacher development, student assessment, and institutional self-evaluation (9). The College of Medicine at Qassim University exhibited improved quality oversight and student learning following the completion of a comprehensive accreditation cycle (10). Similarly, pharmacy institutions indicated improved curriculum integration, stakeholder involvement, and the establishment of internal quality assurance procedures following accreditation (11).

However, the implementation and sustaining of accreditation pose significant challenges. Colleges must establish systems to perpetually gather, evaluate, and respond to data across several disciplines. They must also cultivate faculty endorsement and modify organizational frameworks to facilitate accountability and performance assessment (12, 13). Despite these obstacles, the advantages of accreditation—namely recognition, enhanced outcomes, and academic credibility—persist in motivating pharmacy institutions to allocate resources towards quality assurance. Moreover, quantitative instruments such as the Analytic Hierarchy Process (AHP) and Multi-Objective Optimization by Ratio Analysis (MOORA) support evidence-based curriculum improvement by offering systematic, transparent approaches for prioritizing educational objectives and assessing Program Learning Outcomes (PLOs) achievement over time. Traditional assessments of Program Learning Outcomes (PLOs) in Saudi pharmacy institutions are often disjointed, retrospective, and inadequate in facilitating ongoing enhancement. They frequently lack integration with stakeholder feedback and do not facilitate year-over-year tracking necessary for accreditation and curriculum renewal. The AHP-MOORA framework mitigates this significant deficiency by providing a systematic, stakeholder-informed methodology that facilitates longitudinal monitoring of graduating cohorts. It enables dynamic benchmarking of outcome achievement, connecting educational performance with changing national competency standards. This dual-function design promotes internal quality assurance and strengthens preparedness for national and international accreditation processes.

Despite the emphasis on accreditation and learning outcome assessment, most existing models lack structured integration of stakeholder priorities and do not enable longitudinal performance tracking across multiple cohorts. This study addresses that gap by implementing a dual-framework monitoring model for Pharm.D PLOs achievement at King Khalid University’s College of Pharmacy. By combining Analytic Hierarchy Process (AHP) and Multi-Objective Optimization by Ratio Analysis (MOORA), and aligning it with NCAAA standards and national priorities, the model offers a replicable approach for continuous quality improvement in pharmacy education.

## Methods

### Study design and context

This research utilized a systematic multi-criteria decision-making methodology to establish and execute a monitoring framework for evaluating the attainment of PLOs in the Pharm.D program at a public pharmacy college in Saudi Arabia. The architecture reflects decision-analytic methodologies employed in engineering accreditation systems while being tailored to the pharmaceutical domain and the criteria set by the National Commission for Academic Accreditation and Assessment (NCAAA) (1).

### PLO determination and framework alignment

The PLOs were formulated based on the Pharm.D program PLOs framework, which corresponds with the Saudi Arabian Qualifications Framework (SAQF) and the five areas of the NCAAA: knowledge, cognitive skills, interpersonal skills and responsibility, communication and IT skills, and psychomotor abilities. This study adopted the full set of eight PLOs as defined by the institutional Pharm.D curriculum. These PLOs align with the SAQF and NCAAA competency domains and are listed with their quantitative indicators in Table 1.

**Table 1:** Quantitative indicator mapping for MOORA.

PLO code	PLO description	Quantitative indicator(s) used in MOORA
PLO1	Knowledge of pharmaceutical and biomedical sciences	Cumulative GPA at graduation, performance in licensing exams
PLO2	Clinical application and decision-making	Proportion of graduates employed in clinical pharmacy roles; average scores in clinical clerkship assessments
PLO3	Communication skills	Alumni self-evaluation of communication effectiveness (survey); employer satisfaction ratings on communication (survey)
PLO4	Teamwork and collaboration	Peer evaluation scores from group assignments; preceptor evaluations during teamwork-based training
PLO5	Ethics and professionalism	Number of reported ethics violations per cohort; average scores in ethics/professionalism sections of alumni surveys
PLO6	Digital health competency	Proportion of graduates who used digital pharmacy tools in practice; performance scores in institutional digital competency modules
PLO7	Compounding and dispensing	Practical exam scores from compounding labs; preceptor assessments during dispensing rotations
PLO8	Lifelong learning and development	Proportion of graduates enrolled in postgraduate studies; participation in CPD programs, workshops, or professional conferences

These outcomes were translated into measurable indicators across alumni employment records, postgraduate enrollment, and documented clinical roles.

### Stakeholder engagement and weight assignment (AHP method)

The Analytical Hierarchy Process (AHP) was employed to determine the relative importance of each Program Learning Outcome (PLO). A total of 21 stakeholders participated in the AHP weighting process, including employers, program graduates, academic administrators, and faculty members. Each participant independently completed the pairwise comparisons using Saaty’s 1–9 scale. To ensure the internal consistency of judgments, a consistency ratio (CR) was calculated for each matrix. Only matrices with a CR < 0.1 were accepted, as recommended by AHP methodology standards. The final weights for each PLO were obtained by aggregating the normalized values from all valid matrices.

### AHP formulas and consistency validation

AHP Weight Calculation, which calculates the average normalized value for each PLO across all comparisons. Each panel member completed the AHP pairwise comparisons independently using Saaty’s 1–9 scale (Equation 1).


(1) $$W_{i}=\frac{1}{n}\sum_{j=1}^{n}(\frac{a_{\mathrm{ij}}}{\sum_{k=1}^{n}a_{\mathrm{kj}}})$$

Where: $W_{i}$ : normalized weight for the *i*th Program Learning Outcome (PLO); *n*: total number of PLOs; $a_{\mathrm{ij}}$ : importance score of PLOi compared to PLOj in the pairwise comparison matrix; Σ is column sum of comparisons for PLOj.

AHP Consistency Ratio (CR), calculates the Consistency Ratio (CR), a metric used in the Analytic Hierarchy Process (AHP) to evaluate how consistent the judgments are in a pairwise comparison matrix (Equation 2).


(2) $$\mathrm{CR}=\frac{\mathrm{CI}}{\mathrm{RI}},\;\mathrm{CI}=\frac{\lambda_{\max}-n}{n-1}$$

Where: CR: Consistency Ratio, used to check if judgments are logically consistent; CI: Consistency Index; λmax: Principal eigenvalue of the pairwise comparison matrix; RI: Random Index (average CI of a randomly generated matrix of order n); nn: Number of PLOs; CR value < 0.1 indicates acceptable consistency.

### Mixed-methods integration strategy

This study employed a convergent mixed-methods design, integrating qualitative stakeholder input with quantitative program outcome data. The qualitative component was the Analytic Hierarchy Process (AHP), which used structured pairwise comparisons to collect expert opinions on the relative significance of each PLO. These judgments were synthesized into normalized weights reflecting stakeholder priorities.

The quantitative strand consisted of objective performance indicators linked to each PLO (e.g., employment rates, exam scores, postgraduate enrollment). The Multi-Objective Optimization by Ratio Analysis (MOORA) method was used to process these indicators. Each graduating cohort’s composite achievement score was generated by applying the weights derived from the AHP during MOORA analysis.

This integration allowed for a combined assessment model in which stakeholder-defined priorities directly shaped the evaluation of program outcomes, ensuring both contextual relevance and data-driven rigor.

### Performance evaluation via MOORA

#### Rationale for selecting MOORA

The Multi-Objective Optimization by Ratio Analysis (MOORA) method was used to calculate achievement scores for each PLO across five graduating cohorts (2018–2022) (14). For each year, quantitative indicators (e.g., % employed in clinical roles, % enrolled in postgraduate studies) were normalized and weighted using the AHP-derived scores. Maximization criteria (e.g., postgraduate enrollment) and minimization criteria (e.g., time to first employment) were both integrated according to Brauers and Zavadskas’ method (14). Final composite scores were computed to rank cohort performance longitudinally.

#### Why MOORA is ideal for longitudinal tracking

The MOORA method was selected due to its proven capability in multi-criteria decision-making contexts where multiple, and sometimes conflicting, educational performance indicators must be evaluated simultaneously.

It is especially well-suited for evaluating longitudinal programs because of its structure, which permits the inclusion of both cost-based (like time to employment) and beneficial (like postgraduate enrollment) criteria. By using stakeholder-defined weights from the AHP process and normalizing all indicators to guarantee comparability across units and scales, trade-offs between better and worse-performing outcomes were addressed.

Each graduating cohort’s performance was evaluated using the MOORA method, incorporating both beneficial and cost-based indicators across five graduating years (2018–2022). Cohort performance indicators (e.g., employment rate, postgraduate enrollment, clinical role) were collected for 2018–2022. The decision matrix was normalized, which was calculated by Equation 3: MOORA normalization formula.


(3) $$X_{\mathrm{ij}}^{*}=\frac{X_{\mathrm{ij}}}{\sqrt{\sum_{i=1}^{n}X_{\mathrm{ij}}^{2}}}$$

Where, $X_{\mathrm{ij}}^{*}$ normalized score for cohort ii on criterion *j*; $X_{\mathrm{ij}}$ : Raw score of cohort i for criterion *j*; *n*: number of cohorts; ∑ $X_{\mathrm{ij}}^{2}$ : sum of squared scores for criterion *j* across all cohorts. This equation standardizes all scores to eliminate scale differences.

Each cohort’s total performance score yi was computed by using the MOORA composite score calculation (Equation 4).


(4) $$Y_{i=}\sum_{j=1}^{g}X_{\mathrm{ij}}^{*}-\sum_{j=g+1}^{n}X_{\mathrm{ij}}^{*}$$

Where, yi: overall MOORA score for cohort ii; $X_{\mathrm{ij}}^{*}$ : normalized score for cohort ii on criterion j; g: number of beneficial criteria; *n*: total number of criteria (beneficial + cost). The formula subtracts less desirable (cost) indicators from beneficial ones to calculate a net score.

#### Quantitative indicator mapping for MOORA

To operationalize the eight PLOs for use in MOORA analysis, each was linked to specific and measurable performance indicators. These indicators were derived from institutional records, survey responses, and experiential assessments. The alignment between each PLO and the associated indicator or indicators is shown in Table 1.

### Monitoring tool development

A solution based on Microsoft Access was created to collect alumni data, implement AHP weights, normalize numbers using MOORA, and illustrate annual trends. The database had tables for graduate profiles, career information, postgraduate study records, and clinical positions. Alumni statistics were updated each year using a synthesis of institutional records, LinkedIn, employment verification, and self-reported surveys.

### Ethical considerations

The study received ethical clearance from the Research Ethical Committee at King Khalid University (approval no. KKU-14-2025-10). Alumni data were anonymized, and all participants in surveys or interviews provided informed consent.

## Results

### Stakeholder characteristics

A total of 21 stakeholders participated in the AHP pairwise weighting process. Their demographic and professional distribution is summarized in Table 2. Participants represented key roles relevant to curriculum delivery, program oversight, and graduate employment. All contributors had direct experience with Pharm.D program evaluation and at least 3 years of professional practice in their respective domains.

**Table 2:** Participant demographics.

Characteristics	*n*(%)
Gender	
Male	12 (57.1%)
Female	9 (42.9%)
Stakeholder role	
Faculty members	8 (38.1%)
Alumni	6 (28.6%)
Employers	4 (19.0%)
Academic administrators	3 (14.3%)
Years of experience (range)	
7–18 years	8 (38.1%)
3–8 years	6 (28.6%)
10–20 years	4 (19.0%)
6–15 years	3 (14.3%)
Typical background	
Pharmacy education and clinical instruction	8 (38.1%)
Recent graduates in community/hospital roles	6 (28.6%)
Hospital directors, pharmacy supervisors	4 (19.0%)
Curriculum directors, QA coordinators	3 (14.3%)

Stakeholders were selected based on involvement in curriculum delivery, program evaluation, or graduate employment. All had a minimum of 5 years of experience in pharmacy education or practice.

### Stakeholder prioritization of PLOs using AHP

A panel of 21 stakeholders, including academics, graduates, and employers, engaged in pairwise comparisons of PLOs utilizing the Analytic Hierarchy Process (AHP). Their contributions determined the significance of each PLO in alignment with curricular aims and the requirements of the Saudi workforce. PLO2 (clinical application) was assigned the most weight (0.28), followed by PLO5 (ethics and professionalism, 0.19) and PLO1 (pharmaceutical/biomedical knowledge, 0.17). The outcome with the lowest weight was PLO8 (lifelong learning, 0.04), indicating its more prolonged, rather than immediate, effect on early-career performance (Table 3).

**Table 3:** AHP-derived weights for program learning outcomes.

PLO	Description	Weight
PLO2	Clinical application	0.28
PLO5	Ethics and professionalism	0.19
PLO1	Biomedical/pharmaceutical knowledge	0.17
PLO4	Teamwork and collaboration	0.11
PLO3	Communication	0.09
PLO6	Digital health integration	0.07
PLO7	Compounding and dispensing	0.05
PLO8	Lifelong learning	0.04

**Figure 1:**
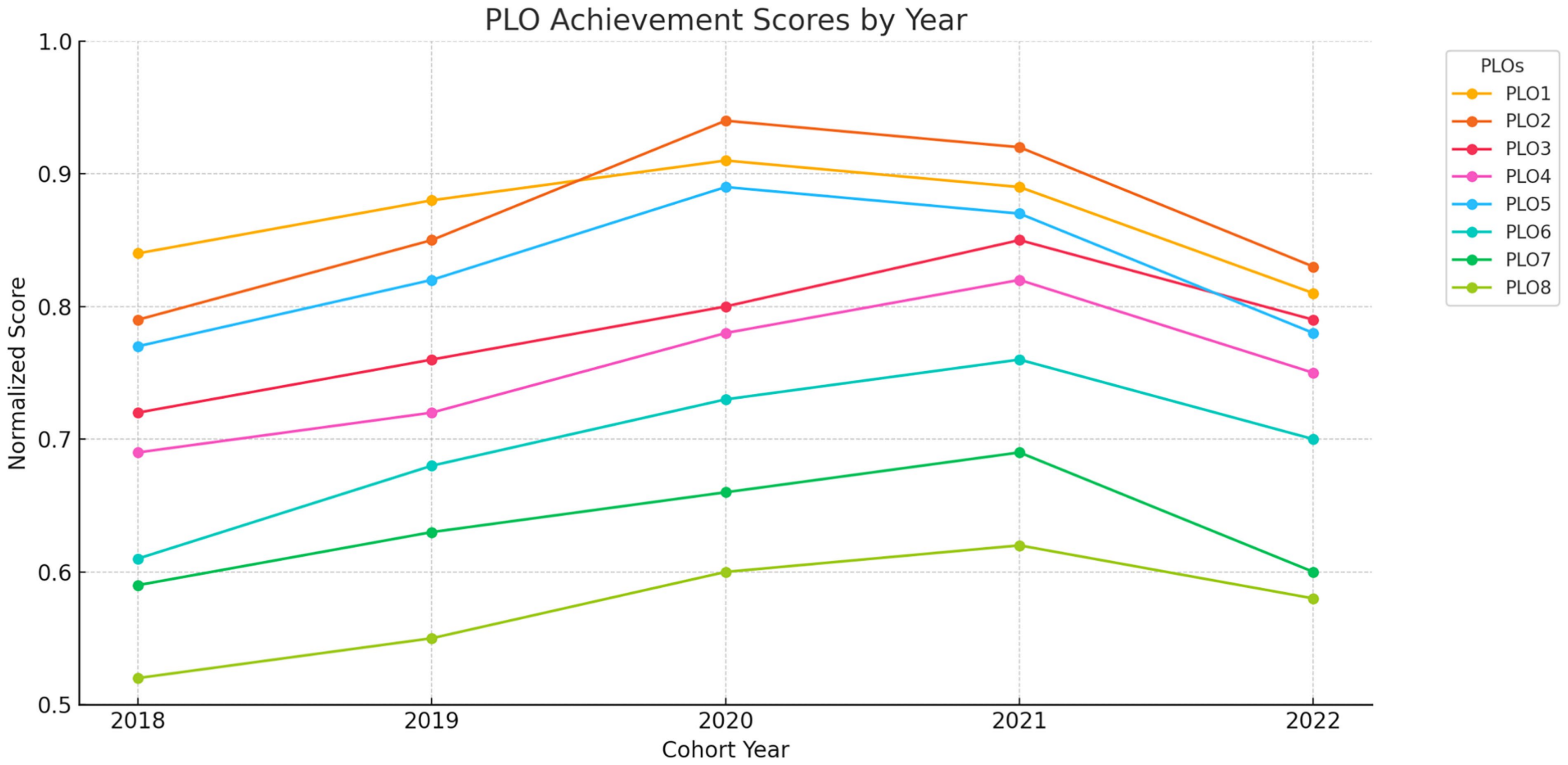
PLO achievement scores by year. Normalized MOORA scores for each PLO across five cohorts (2018–2022).

The stakeholder priorities guided the weighting of performance data across cohorts, facilitating an outcome-oriented and relevance-based comparative framework. Figure 1 illustrates a summary of PLO performance patterns throughout cohorts, indicating that PLO2 and PLO5 not only have the greatest weights but also consistently attained superior scores in real cohort achievement.

### MOORA-based cohort performance

Normalized scores for all eight PLOs were calculated across five graduating cohorts from 2018 to 2022 using Multi-Objective Optimization by Ratio Analysis (MOORA). The scores were derived from quantitative metrics including postgraduate enrollment, job placement, and positions in patient care. The findings indicated that the cohorts of 2020 and 2021 consistently attained the best PLOs performance scores, especially in PLO2 and PLO5, demonstrating strong clinical integration and ethical training throughout those years (Table 4).

**Table 4:** Normalized PLO scores across graduating cohorts.

PLO	2018	2019	2020	2021	2022
PLO1	0.84	0.88	0.91	0.89	0.81
PLO2	0.79	0.85	0.94	0.92	0.83
PLO3	0.72	0.76	0.80	0.85	0.79
PLO4	0.69	0.72	0.78	0.82	0.75
PLO5	0.77	0.82	0.89	0.87	0.78
PLO6	0.61	0.68	0.73	0.76	0.70
PLO7	0.59	0.63	0.66	0.69	0.60
PLO8	0.52	0.55	0.60	0.62	0.58

**Figure 2:**
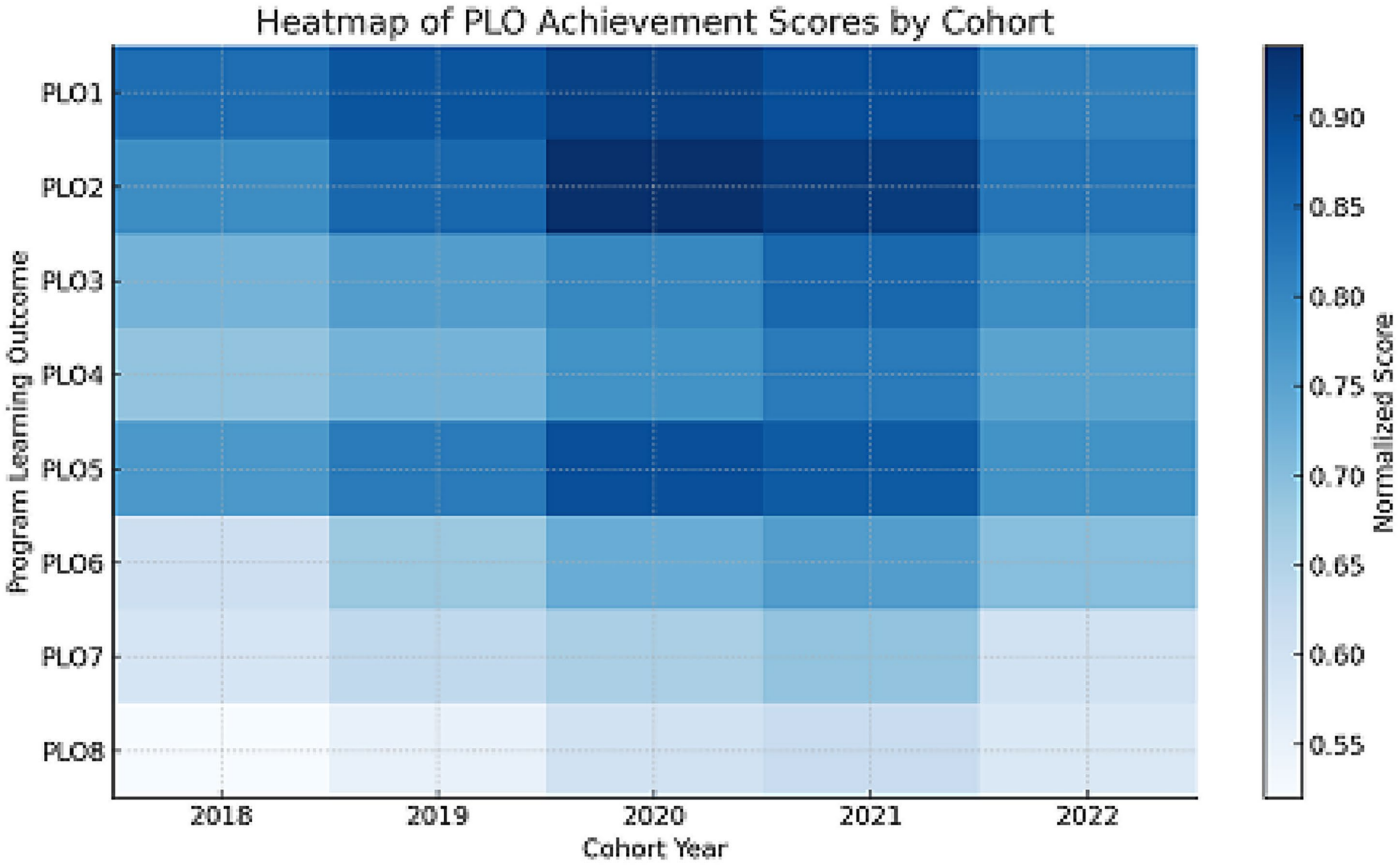
Heatmap of PLOs achievement scores by cohort. Visual density of normalized PLOs scores by cohort; red = high performance, blue = low.

The chart highlights improvement over time in critical PLOs such as PLO3 (communication) and PLO4 (teamwork), and flags consistent underperformance in PLO8 (Figure 2).

### Composite rankings and longitudinal trends

Composite cohort performance was ranked after adding stakeholder-derived AHP weights to MOORA-normalized scores. The 2020 cohort attained the greatest score (0.883), followed by the 2021 cohort (0.871); however, the 2022 cohort recorded the lowest composite score (0.792), indicating potential instructional deficiencies or external disturbances (Table 5).

**Table 5:** Composite MOORA scores and cohort rankings.

Cohort	Weighted score	Rank
2020	0.883	1
2021	0.871	2
2019	0.836	3
2018	0.811	4
2022	0.792	5

**Figure 3:**
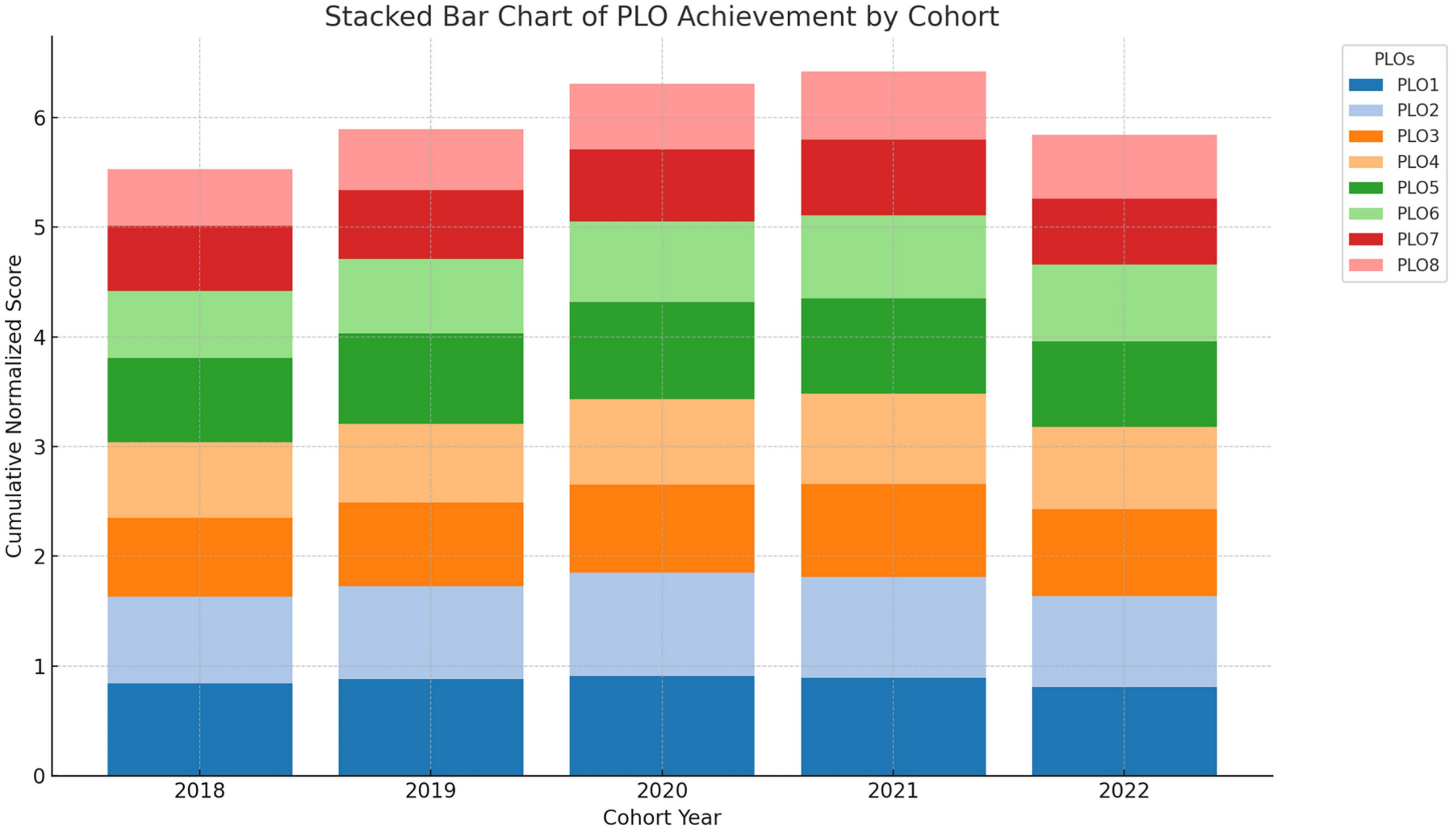
Stacked bar chart of PLO contributions. Cumulative impact of each PLO per cohort, illustrating weight distribution and relative emphasis.

**Figure 4:**
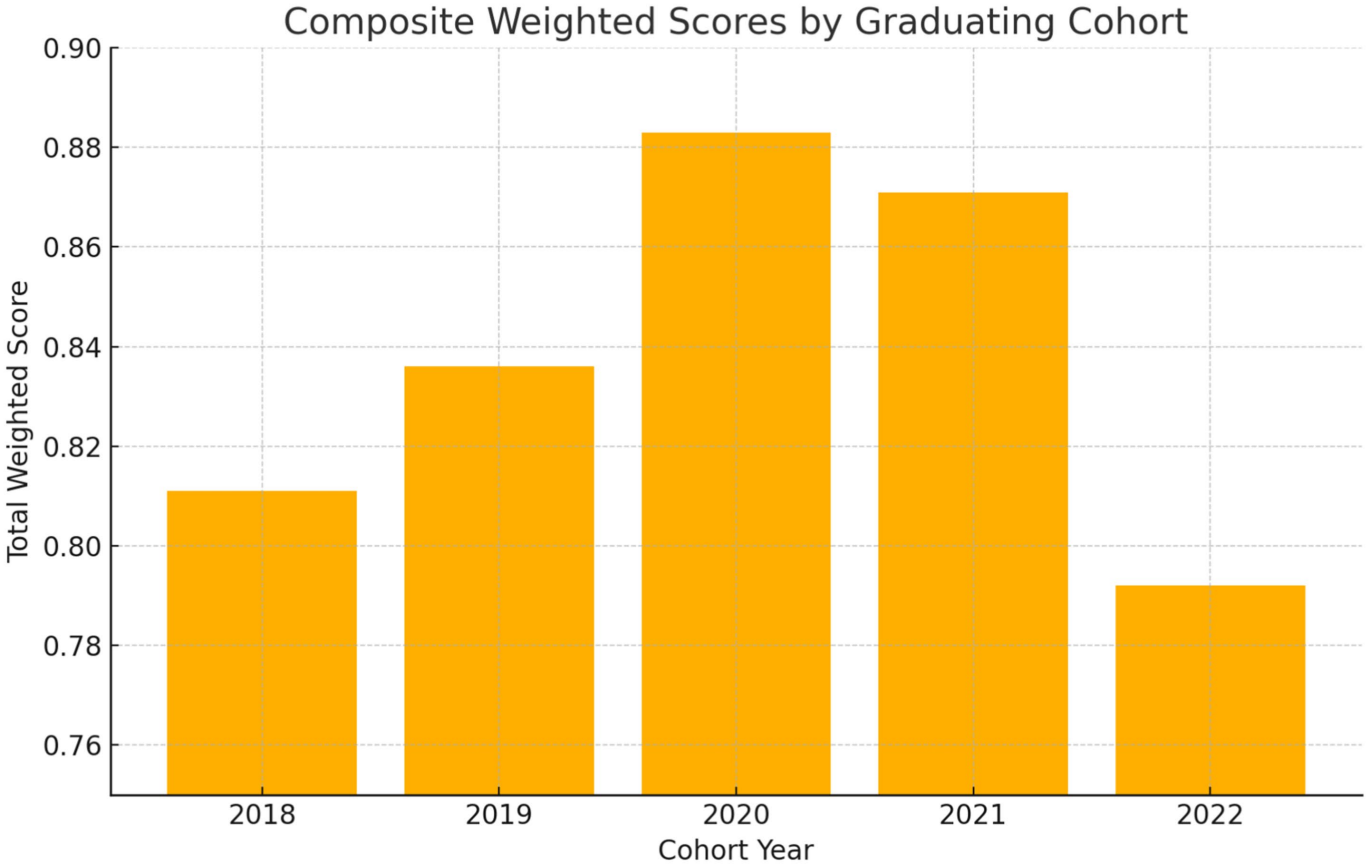
Summary of PLOs achievement scores across cohorts. Multi-line comparison of PLOs scores across five years.

Figure 3 illustrates a stacked bar chart depicting the contributions of the PLOs to enhance comprehension of the composite cohort development. The uniformity in high-performing PLOs, such as PLO2 and PLO5, is visually apparent, but underperforming areas like PLO6 (digital health) necessitate targeted enhancement. Figure 4 presents a longitudinal summary graphic that highlights performance changes across years, distinctly illustrating peak outcome scores in 2020 and 2021, followed by a relative fall in 2022.

Figure 4 provides a comparative visualization of PLOs achievement trends across five graduating cohorts, illustrating longitudinal performance patterns by outcome.

## Discussion

### Overview of the AHP-MOORA framework

This study introduced a dual-framework monitoring system using the Analytic Hierarchy Process (AHP) and Multi-Objective Optimization by Ratio Analysis (MOORA) to evaluate the longitudinal achievement of PLOs in a Saudi Pharm.D program. The tool was designed to meet national accreditation requirements set by the National Commission for Academic Accreditation and Assessment (NCAAA) while promoting internal quality assurance and responsiveness to stakeholder priorities.

The AHP-MOORA framework, utilized for the longitudinal assessment of Pharm.D Program Learning Outcomes (PLOs) at King Khalid University’s College of Pharmacy, provides a comprehensive, data-driven instrument for aligning educational outcomes with National Commission for Academic Accreditation and Assessment (NCAAA) standards (6, 10). By employing the Analytical Hierarchy Process (AHP) for stakeholder prioritization and Multi-Objective Optimization by Ratio Analysis (MOORA) for cohort performance evaluation, the framework exceeds conventional subjective assessments, delivering accurate, actionable insights for accreditation and quality assurance (14, 15). The Microsoft Access-based solution facilitated effective data management, indicating peak cohort performance in 2020 (0.883) and a decrease in 2022 (0.792) across five cohorts (2018–2022). This model’s conformity with NCAAA’s continuous quality improvement requirements and Saudi Arabia’s Vision 2030 healthcare objectives establishes it as a scalable solution for pharmacy education, potentially improving accreditation preparedness across Saudi and Gulf Cooperation Council (GCC) institutions (1, 2, 4).

### Stakeholder priorities and PLOs emphasis

The AHP results ranked PLO2 (clinical application, 0.28) and PLO5 (ethics and professionalism, 0.19), indicating stakeholder prioritization of patient-centered and ethical competencies, in alignment with Mutalib et al.’s findings regarding the significance of technical and professional skills in engineering education, which have been adapted to the clinical focus of pharmacy (16). These priorities correspond with the Saudi Arabian Qualifications Framework (SAQF) and the global CanMEDs framework, which underscore values-based practice, although they diverge from Aljadhey et al.’s emphasis on research skills (PLO7) in certain Saudi programs (3, 17, 18). The minimal emphasis on PLO8 (lifelong learning, 0.04) deviates from the Accreditation Council for Pharmacy Education (ACPE) criteria, indicating a regional prioritization on urgent workforce preparedness, as observed in Mokhtar’s GCC study (4, 19).

### Cohort trends and performance patterns

MOORA scores demonstrated exceptional performance in PLO2 and PLO5, particularly in 2020 and 2021 (e.g., PLO2: 0.94 in 2020), signifying effective clinical and ethical training, supported by Alshahrani’s study on enhancements in quality assurance within Saudi pharmacy programs (7). Nevertheless, ongoing underachievement in PLO6 (digital health integration, 0.70 in 2022) and PLO7 (compounding and dispensing, 0.60 in 2022) underscores deficiencies in technical and practical competencies, corresponding with national digital transformation obstacles (2, 4).

The 2020 cohort achieved a high performance of 0.883, whereas the 2022 cohort had a fall to 0.792, indicative of external factors, particularly the disruptions caused by COVID-19. Alrushud et al. showed diminished academic engagement among Saudi medical students during lockdowns (19). Conversely, Al Mohaimeed et al.’s investigation of Saudi medical education revealed consistent post-accreditation results, indicating that pharmacy programs encounter distinct difficulties in sustaining experiential learning throughout crises (7).

### Benchmarking and literature comparison

The longitudinal trend visualization of the Microsoft Access tool surpassed the manual techniques outlined by Khojah and Shousha and aligned with the data collecting system of Mutalib et al. for engineering outcomes; however, its AHP-driven stakeholder integration provides distinct customization (12, 16). In contrast to Malik et al.’s global quality assurance paradigm, the AHP-MOORA framework demonstrates superior stakeholder participation and data-driven decision-making, hence improving accountability (20). These findings highlight the necessity for focused interventions, such as virtual simulations and telehealth training, to rectify weaknesses in PLO6 and PLO7 and maintain performance during disruptions. Comparable outcome-based evaluation frameworks in pharmacy and engineering education have illustrated the effectiveness of systematic assessment instruments. Elkhalifa et al. (17) identified deficiencies in competency evaluation within pharmacy graduate programs, underscoring the necessity for a standardized Program Learning Outcomes tracking system such as AHP-MOORA. Similarly, Wu et al. (8) applied AHP in the assessment of student performance, emphasizing its significance in the oversight of higher education.

### Internal quality improvement applications

Beyond external accreditation, the AHP-MOORA model also serves as a robust mechanism for internal quality improvement. Pharmacy colleges can use the tool to support annual curriculum reviews, departmental performance audits, and stakeholder feedback integration. By tracking PLO attainment trends over time, faculty can identify underperforming domains and implement targeted revisions. Furthermore, the model supports real-time reporting to academic boards, enhances transparency during program self-studies, and facilitates continuous dialogue among faculty, administrators, and external partners. Consistently reduced MOORA results in PLO domains, including digital health and pharmaceutical compounding, necessitated internal task force assessments and course redesigns. These findings endorse focused teacher development and improved evaluation instruments aligned with performance gaps.

### Limitations and implications

This study possesses a few limitations that need to be acknowledged when evaluating the results. The investigation was restricted to a singular public pharmacy institution in Saudi Arabia, potentially constraining its applicability to other institutional contexts or educational frameworks. The limited number of stakeholders participating in the AHP weighing process (*n* = 21) may inadequately represent various points of view of academics, alumni, and employers. Furthermore, although stakeholder involvement was integral to the AHP process, it may have generated subjective bias in the prioritization of learning objectives.

Data reliability is an additional factor to consider. Segments of the data—especially postgraduate enrollment and alumni job status—were self-reported and prone to recall bias or incompleteness. Despite the enhancement of accuracy through cross-validation using employment documents, LinkedIn profiles, and institutional data, deviations may persist, particularly among graduates employed abroad or from earlier cohorts.

Technical constraints also took place in the application of Microsoft Access for MOORA computation. The platform was selected for its institutional familiarity and ease of deployment; nevertheless, scalability may be limited for larger programs or cross-institutional applications. Nonetheless, the framework’s architecture facilitates prospective integration with automated analytics, as proposed by Alhakami et al. (21) and Mutalib et al. (16), potentially alleviating faculty effort and enhancing real-time responsiveness.

Notwithstanding these constraints, the AHP-MOORA framework exhibits robust conformity with both national (NCAAA, SAQF) and international (ABET) certification standards. This model incorporates structured prioritizing and longitudinal cohort tracking, improving its effectiveness for academic quality assurance compared to previous ABET-aligned methods. Al Mohaimeed et al. (7) asserts that teacher stress and workload continue to impede effective evaluation processes. Nonetheless, institutions implementing specialist teams, like to those delineated by Shaiban (22) in the realm of medical education, may alleviate these issues.

The extensive implementation of this approach at Saudi pharmacy universities, alongside a comparison with ACPE-accredited programs, may confirm its scalability and flexibility. The paradigm enables outcome-focused program assessment and curriculum development, particularly in digital health, resource distribution, and faculty engagement, offering a sustainable method for continuous quality advancement in pharmacy education (21, 23).

Quantitative decision-making tools like AHP and MOORA are essential for promoting evidence-based curriculum enhancement by providing organized, transparent, and replicable techniques for prioritizing educational objectives and monitoring the achievement of learning outcomes across cohorts. These approaches augment institutional capability to analyze intricate performance data, facilitate informed curricular modifications, and synchronize academic outcomes with national competency benchmarks. Their structured framework enables longitudinal assessment and enhances qualitative stakeholder feedback, thereby adhering to the principles of mixed-methods implementation research, which emphasizes the combination of quantitative precision and contextual insight to connect evidence with practice in actual educational environments (24).

### Future directions

Future studies should examine whether this framework can be applied to other Pharm.D programs in Saudi Arabia and abroad. Validating the model in various regulatory contexts may be aided by comparative assessments with ACPE-accredited institutions. Technological improvements such as cloud-based dashboards and automated analytics are recommended to support scalability. It will also be crucial to improve performance indicators for underdeveloped domains (such as ethics and digital competency) and encourage continued faculty participation in quality monitoring to ensure long-term adoption and sustainability.

## Conclusion

This study established and executed a comprehensive, stakeholder-informed framework employing the Analytic Hierarchy Process (AHP) and Multi-Objective Optimization by Ratio Analysis (MOORA) to assess PLOs in a Saudi Pharm.D program. The methodology effectively linked institutional performance to the national accreditation standards set by the National Commission for Academic Accreditation and Assessment (NCAAA), as well as the overarching objectives outlined in Vision 2030 and the Saudi Arabian Qualifications Framework (SAQF). By involving stakeholders in the prioritization process, the AHP technique guaranteed that curriculum review was pertinent and aligned with national workforce requirements. Simultaneously, MOORA facilitated normalized and data-driven comparisons among graduating cohorts, promoting transparent performance analysis over a five-year duration. The results indicated significant success in clinically oriented and morally motivated outcomes, especially in 2020 and 2021, but also revealed ongoing deficiencies in digital health and compounding competencies. The framework’s scalability, real-time dashboard capabilities, and compliance with accreditation criteria render it an efficient internal quality assurance instrument. It enables schools to convert performance data into curricular improvements, pinpoint areas needing remediation, and proactively prepare for external assessment. In conclusion, the AHP-MOORA model provides a systematic and reproducible methodology for evaluating PLOs. Its implementation can foster enduring academic excellence and assist pharmacy programs in achieving evolving national and international standards for competency-based education.

The AHP-MOORA model offers a transferable framework that can be modified for pharmacy education programs in other regional and global contexts, going beyond the current institutional and national scope. Its design enables adaptable alignment with regional workforce priorities, cultural contexts, and accreditation requirements. Institutions around the world can modify the model to support their internal quality assurance procedures while staying consistent with more general competency-based educational standards by modifying stakeholder selection and performance metrics. This strategy can be implemented across pharmacy colleges in Saudi Arabia and the Gulf area with minor adjustments to stakeholder panels and institutional metrics. Its alignment with NCAAA and SAQF frameworks guarantees extensive applicability for accreditation, curriculum benchmarking, and regional quality assurance.

## Data Availability

The raw data supporting the conclusions of this article will be made available by the authors, without undue reservation.
